# 

**Published:** 1858-04

**Authors:** 


### NOTICES, &c.

TUCKER'S *Annual Register*, for 1858, Albany, N. Y. LUTHER TUCKER & Son. Sent for 25 cts., 124 pp., 130 engravings. Affords more useful information for a farmer than can be anywhere procured for an equal amount of money. And if any farmer had to lose one meal a day for a week, to save a dollar to purchase the vols. for 1855, 6, and 7, bound in one vol., and sent for that dollar, he and his family would be life-long gainers—440 engravings, treating of over 500 different subjects, in reference to gardening, farming, and domestic appliances. We believe it to be the most variously useful book to all persons who live in the country, we have ever noticed.

*Presbyterian Expositor*, Chicago — edited by Rev. N. L. RICE, D.D., $1.50 a year—is, we believe, the only publication in this country, or the world, the express object of which is to define *Presbyterianism;* and it largely merits the patronage of that intelligent, energetic, and liberal denomination. We suggest that the month and number of the issue be placed at the top of the first page of the cover. We have received only the first two numbers. By all means let a list of contents be placed on the last page of the cover.

AN Essay on the *Preservation of the Health*, by GOODWIN NIXON, M.D., of Lowndes, Ala., with the motto "not merely to live, but to live well," that is, in good health, contains many excellent suggestions.

DR. DAVIS, a member of the Massachusetts Medical Society, has published a useful tract on Special Exercises in the treatment of DISEASES OF THE LUNGS. It is an effort in the right direction, and foreshadows a wiser practice in the treatment and cure of consumptive disease. No medicine ever swallowed is curative of the malady; we can hope to master it only by proper activities.

*Lectures to Children on the Bible*, by Rev. S. G. GREEN, B. A., of New Brunswick, in *The Religious Intelligencer*, of St. John's; also several articles in *The New York Observer*, containing Prayers and Thanksgiving in the language of Scripture, we heartily commend to public notice. Forty years ago, the most of a prayer was in Scripture language; let there be a return to the good old custom. It is not the least beneficial result of the Episcopal Prayer Book, that its frequent use of the language of the Bible keeps us from wandering, and reins us in to truth and reality; it largely helps, as ought to be done, to make the Bible and Bible incidents classic ground to the children of the Church.

IN an Annual Address, delivered before the Medical Society of North Carolina, Dr. S. S. SATCHWELL, of New Hanover county, has presented some views in a forcible light on "Obstacles to Medical Progress," which are well worthy of the mature consideration of the Medical Profession.

W. W. ALLPORT, Dentist, of Chicago, read a paper before the Cook County Medical Society, on *Diseases of the Teeth*, which, for its practical truthfulness, the Society deemed worthy of publication. It is by the diffusion of tracts like this, which instruct the people, that professional men must hope to add to their business and to do good besides. THE BEST INFORMED PATRONIZE DENTISTS MOST. The best informed soonest apply to the physician, and are the last to tamper with their health, their constitution, and their lives.

*Eclectic Medical Journal*, Cincinnati. The last number contains a variety of excellent articles.

*The Law of Liberality* between Christian Denominations, applied to the Protestant Episcopal Church, by a clergyman of Cincinnati, Ohio, reprinted by the liberality of a young gentleman of New York, is a tract by a "non-exclusive Churchman," the object of which is "to promote greater unity among all Evangelical Denominations, by removing what he deems erroneous impressions respecting his own."

This tract will do good, for the more plainly and truly any orthodox society presents its distinctive features, the sooner will it be found there is no very great difference after all, that the true-hearted of every Church are humbly aiming at the same mark, striving for the same victory, the victory over sin and death, and hoping for the same reward, a resting-place in the bosom of the Father in Heaven.

*Graham's Magazine*, for April, opens with a most interesting and instructive article by JOSEPH J. REED, on the "Black Death," a disease which, in the middle of the fourteenth century, destroyed, according to the estimates, not less than *twenty-five millions* of persons. The great truth presented is, that its worst ravages were preceded by great drouth or great floods. *Graham* has of late increased the value of its pages, by solid articles of practical, scientific, and historical interest

